# Understanding multifactorial influences on the continuum of maternal weight trajectories in pregnancy and early postpartum: study protocol, and participant baseline characteristics

**DOI:** 10.1186/s12884-015-0490-7

**Published:** 2015-03-28

**Authors:** Tiffany A Moore Simas, Silvia Corvera, Mary M Lee, NingNing Zhang, Katherine Leung, Barbara Olendzki, Bruce Barton, Milagros C Rosal

**Affiliations:** 1 Department of Obstetrics & Gynecology, Division of Research, University of Massachusetts Medical School/UMass Memorial Health Care, Memorial Campus – 119 Belmont Street, Worcester, MA 01605 USA; 2 Department of Medicine, Program in Molecular Medicine, University of Massachusetts Medical School, Biotech 2 – 373 Plantation Street, Worcester, MA 01605 USA; 3 Department of Pediatrics, University of Massachusetts Medical School/UMass Memorial Health Care, University Campus – 55 Lake Avenue, Worcester, MA 01655 USA; 4 Department of Medicine, Division of Preventive and Behavioral Medicine, University of Massachusetts Medical School, University Campus – 55 Lake Avenue, Worcester, MA 01655 USA; 5 Department of Quantitative Health Sciences, University of Massachusetts Medical School, University Campus – 55 Lake Avenue, Worcester, MA 01655 USA

**Keywords:** Design, Methods, Biopsychosocial, Pregnancy, Postpartum, Weight gain, Weight loss, Weight retention, Maternal and child health, Well being

## Abstract

**Background:**

Maternal and offspring immediate and long-term health are affected by pregnancy weight gain and maternal weight. This study was designed to determine feasibility of: 1) recruiting a socio-economically and racially/ethnically diverse sample of pregnant women into a longitudinal observational study, including consenting the women for serial biologic specimen evaluations; 2) implementing comprehensive assessments (including biologic, anthropometric, behavioral, cognitive/psychosocial and socio-demographic, and cultural measures) at multiple time points over the study period, including collecting biologic specimens at planned and unplanned pregnancy delivery times; and 3) retaining the sample for one year into the postpartum period. Additionally, the study will provide preliminary data of associations among hypothesized predictors, mediators and moderators of pregnancy and post-partum maternal and infant weight trajectories. The study was conceptualized under a Biopsychosocial Model using a lifespan approach. Study protocol and baseline characteristics are described.

**Methods/Design:**

We sought to recruit a sample of 100 healthy women age 18–45 years, between 28–34 weeks gestation, with singleton pregnancies, enrolled in care prior to 17 weeks gestation. Women provide written consent for face-to-face (medical history, anthropometrics, biologic specimens), and paper-and-pencil assessments, at five time points: baseline (third trimester), delivery-associated, and 6-weeks, 3-months and 6-months postpartum. Additional telephone-based assessments (diet, physical activity and breastfeeding) administered baseline and three-months postpartum. Infant weights are collected until 1-year of life. We seek to retain 80% of participants at six-months postpartum and 80% of offspring at 12-months.

110 women were recruited. Sample characteristics include: mean age 28.3 years, BMI 25.7 kg/m^2^, and gestational age at baseline visit of 32.5 weeks. One-third of cohort was non-white, over a quarter were Latina, and almost a quarter were non-US born. The cohort majority was multigravida, had graduated high school and/or had higher levels of education, and worked outside the home.

**Discussion:**

Documentation of study feasibility and preliminary data for theory-driven hypothesis of maternal and child factors associated with weight trajectories will support future large scale longitudinal studies of risk and protective factors for maternal and child health. This research will also inform intervention targets facilitating healthy maternal and child weight.

## Background

Maternal weight before, during, and after pregnancy is of considerable public health importance given its impact on both immediate and long-term maternal and child health. The majority of women and thus their children are at weight-related health risk when considering: (1) two-thirds of American women are overweight or obese [1-4], with nearly 50% entering pregnancy overweight or obese, and one in five obese [5], (2) up to 84% of overweight and 74% of obese pregnant women gain above recommended guidelines [6], (3) up to two thirds of women retain weight after pregnancy [7], and (4) some women continue to gain weight rather than lose weight in the postpartum period [8]. Excessive gestational weight gain contributes to subsequent postpartum weight retention [7], and failure to lose pregnancy weight by six months postpartum predicts long-term obesity [9,10] with women retaining an average of 3 kg per pregnancy at 10 years [11]. Given well-established associations between obesity and an array of chronic maternal health conditions, including cardiometabolic diseases [12-15] and some cancers [16], achieving a healthy weight gain during pregnancy, and preventing postpartum weight retention, are critical to the long-term health of many women [9,10].

Maternal obesity is associated with increased risks of gestational diabetes, large for gestational age neonates, and childhood obesity [17]. Excessive maternal weight gain in pregnancy is also associated with childhood obesity [18] which is subsequently associated with long-term offspring consequences including cardiometabolic and neuro-developmental disorders. The growing body of evidence in support of the Developmental Origins of Health and Disease Hypothesis (DOHaD, the Barker hypothesis) [19] suggests that optimizing maternal health before and during pregnancy is critical for improved outcomes not just for women but also for their children.

Acting upon scientific literature on weight gain patterns before, during, and after pregnancy, and in the context of a life-stage framework [3], the Institute of Medicine (IOM) issued gestational weight gain recommendations in 1999, with revisions in 2009. Current recommendations encourage appropriate gain for women with underweight BMIs (<18.5 kg/m^2^) of 28–40 pounds, normal BMIs (18.5-24.9 kg/m^2^) of 25–35 pounds, overweight BMIs (25.0-29.9 kg/m^2^) of 15–25 pounds, and obese BMIs (≥30 kg/m^2^) of 11–20 pounds [3]. The 2009 IOM report also identified a set of consequences that were potentially causally related to gestational weight gain with postpartum weight retention and cesarean delivery emerging as being most important for maternal health; and small for gestational age neonates, large for gestational age neonates, preterm birth and childhood obesity emerging as the most important consequences for infant health [3].

In an effort to promote these guidelines, several randomized control trials have tested interventions to optimize gestational weight gain; however, such interventions have had limited effectiveness [20-26]. Similarly, randomized intervention trials for post-partum weight loss have been limited in their effectiveness [24,27]. Studies have predominantly intervened on diet and exercise, and evaluated effects of breastfeeding [28-31]. With few exceptions [32], the interventions have lacked theoretical grounding and have been limited in the potential array of variables investigated.

The general non-pregnant obesity and weight gain literature has broad comprehensive models for understanding weight and obesity. For example, there has been increased attention to the roles and interplay of reduced sleep [33-35], increased stress [36-38] and depression, all factors that fluctuate during pregnancy and the post-partum period [39-42]. However, examination of these factors has received limited research attention with regards to pregnancy and post-partum weight. In addition to psychosocial factors, biologic considerations include differences in the deposition and mobilization of peripheral and central subcutaneous adipose tissue in the non-pregnant, pregnant and lactating states [43-46].

Given inadequate literature in these and relevant areas, action items in the 2009 IOM report included a recommendation to conduct comprehensive studies that examine how dietary intake, physical activity, dieting practices, food insecurity and, more broadly, the social, cultural and environmental context affect gestational weight gain [3] in large and diverse populations of women. Gestational weight gain and postpartum weight are part of a continuum that needs to be considered in identifying risk factors and mechanisms of postpartum weight retention. Such knowledge is critical to the design of future interventions that have the potential to substantially improve maternal and consequently, child health.

**Figure 1:**
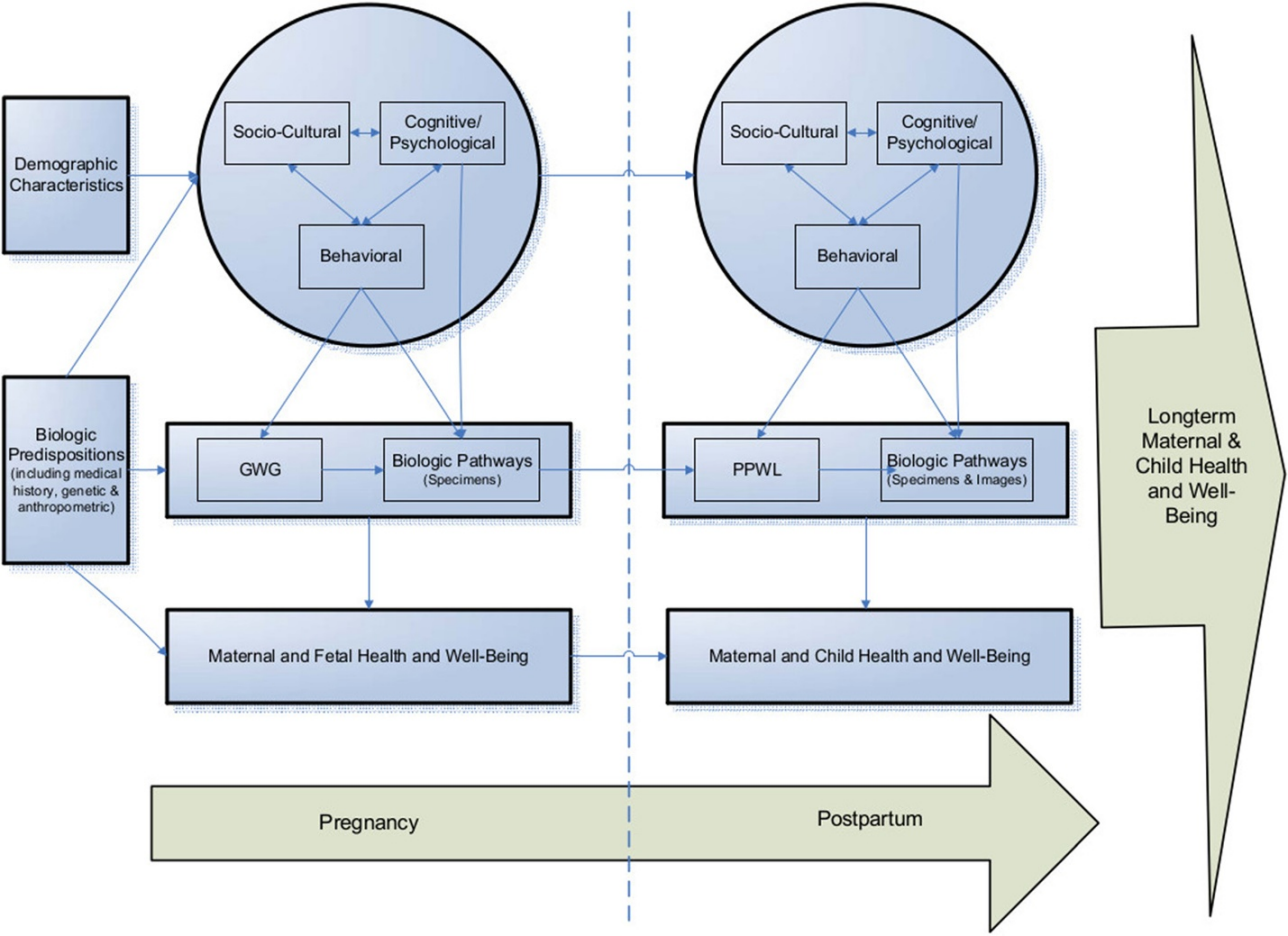
**Transtheoretical framework based on lifespan biopsychosocial model.**

A lifespan approach to the Biopsychosocial Model [47] provides a comprehensive template for examining how the complex interactions among an individual’s biological, psychological and contextual factors affect weight and health outcomes during the childbearing years and beyond, for both the mother and the child. In the current study we conceptualize women’s weight trajectory during both the pregnancy and postpartum periods in addition to long-term maternal and child health and well-being within this biopsychosocial framework (Figure 1). Our study was designed to understand multifactorial influences on maternal weight trajectories that begin in pregnancy and extend into the postpartum period.

### Study aims

To establish the feasibility of: 1) recruiting a socio-economically and racially/ethnically diverse sample of pregnant women into a longitudinal observational study, including consenting the women for serial biologic specimen evaluations; 2) implementing comprehensive assessments (including biologic, anthropometric, behavioral, cognitive/psychosocial and socio-demographic and cultural measures) at multiple time points over the study period, including collecting biologic specimens at planned and unplanned pregnancy delivery times; and 3) retaining the sample for one year into the postpartum period. Additionally, the study will provide preliminary data of associations among hypothesized predictors, mediators and moderators of pregnancy weight gain and postpartum weight loss and retention, in accordance with the multifactorial model shown in Figure 1. Ultimately, this research will inform a future larger study of risks and protective factors for gestational weight gain and post-partum weight loss, with effective interventions to optimize both. This paper describes the study design, assessment protocols, and baseline demographic characteristics of 110 diverse pregnant women and their babies.

## Methods

### Study design & overview

The Pregnancy and Postpartum Observational Determinants Study (PPODS) is a prospective cohort feasibility study approved by the University of Massachusetts Medical School Institutional Review Board human subjects committee. In accordance with the Lifespan Biopsychosocial Model used as the framework for this study, comprehensive assessments starting in pregnancy and continuing over the postpartum period include demographic, biologic including anthropometric, behavioral, cognitive/psychological and socio-cultural factors hypothesized to influence gestational weight gain (GWG), postpartum weight loss, and clinical outcomes of healthy women and their offspring. At present, study recruitment is complete and participant follow up and analyses are ongoing.

### Study setting and population

The study is being conducted at the ambulatory faculty and resident obstetrical practices at a large tertiary hospital in Central Massachusetts, UMass Memorial Medical Center (UMMMC), in Worcester, MA, where approximately 20 residents, 3 nurse practitioners, and 14 attending faculty obstetricians provide prenatal and postpartum care to ethnically and socioeconomically diverse women. These practices deliver ~1,600 of the ~4, 000 pregnancies that are admitted to UMMMC each year.

Eligibility criteria include: (1) age ≥18 or ≤ 45 years, (2) singleton gestation, (3) English speaking, (4) no history of pre-gestational or current gestational diabetes, (5) no evidence of alcohol or substance abuse, (6) not taking medications that affect weight (anti-hypertensives, hypoglycemics, steroids, second generation anti-psychotics, anti-epileptics and thyroid-related pharmaceuticals), (7) no evidence of HIV, hepatitis, autoimmune disease, eating disorder history, or bariatric surgery history, and (8) prenatal care initiated ≤ 16 weeks and 6 days gestational age. Age of inclusion was restricted as GWG recommendations are somewhat controversial in adolescents and general weight gain increases with advancing age. Multiple gestations were excluded due to differences in GWG recommendations for twins, triplets and higher order multiples. Non-English speaking subjects were excluded due to limited resources. Subjects with diabetes were ineligible due to high potentiality of receiving treatments that affect weight (i.e., selected medications, dietary interventions). Subjects with substance abuse history, with prescriptions for aforementioned medication groups and with disease states listed were excluded due to effects on weight, weight gain and diet and on potential compliance and reliability in the former population. Subjects had to have initiated care in early pregnancy so that weight measurements throughout pregnancy were available for analyses.

### Participant screening and recruitment

Identification of eligible women occurred proactively. Between 24–28 weeks gestation, obstetric providers at study practices carry out universal gestational diabetes screening in all gravidas without pre-gestational type 1 or type 2 diabetes mellitus with a 50 g glucose load followed by plasma glucose determination in the subsequent hour [48]. For tracking purposes, a list of consecutive patients undergoing gestational diabetes screening is generated real-time; this prompted review of associated laboratory results by a trained and supervised study coordinator using a HIPAA waiver and the clinical care team. The study coordinator then reviewed medical records of women without gestational diabetes for additional study inclusion/exclusion criteria. Women meeting study criteria who had an upcoming appointment at a gestational age < 34 weeks were placed on a list of potentially eligible women. Their health care providers were subsequently contacted for permission to approach the women and then a letter was mailed to inform potential participants about the study and that they would be approached at their next face-to-face prenatal visit. To support study recruitment efforts, advertisements were placed in public waiting rooms and examination rooms of the obstetric practices, and their physicians were also encouraged to refer patients. All eligible women who expressed interest then provided written informed consent and were assigned a unique alphanumeric identifier.

### Measurements and assessments

Women were assessed at baseline, at delivery, in the immediate in-patient postpartum period and then postpartum at 6 weeks, 3 months and 6 months. Assessments included demographic, biologic including anthropometric, behavioral, cognitive/psychological and sociocultural evaluations. See Table 1 for the assessment schedule. In recognition of participants’ time commitments, women receive gift card incentives following each assessment. Data collection time points for infants are birth, 3-months, 6 months and at 1 year of age. Additionally, infant weight data are obtained from their pediatricians until one year of life.

**Table 1:** **Schedule of study assessments**

Study assessments		Study time points						
		Baseline*	Delivery	Postpartum				
				0-4d	6 wks	3 mos	6 mos	1 year
**Review study protocol & informed consent**		✓						
**Confirmation inclusion/exclusion criteria**								
**Demographics**		✓						
**Biological**	Medical History	✓						
	Blood Pressure	✓		✓	✓	✓	✓	
	Urine for protein and glucose	✓		✓	✓	✓	✓	
	Height	✓						
	Prepregnancy Weight & BMI	✓						
	Skin Fold Thickness (SFT)	✓		✓	✓	✓	✓	
	Waist, Hip and Arm Circumferences	✓		✓	✓	✓	✓	
	Genetics (Maternal buccal swab, umbilical cord and blood, placenta, adipose tissue^†^)^‡^		✓					
**Behavioral**	24 Hour Dietary and Physical Activity Recalls	✓				✓		
	3-Factor Eating Questionnaire R18-modified	✓		✓	✓	✓	✓	
	Food Craving Inventory	✓		✓	✓	✓	✓	
	Pregnancy and Physical Activity Questionnaire (PPAQ) (modified postpartum)	✓		✓	✓	✓	✓	
	Breastfeeding-Infant Feeding Surveys			✓	✓	✓	✓	✓
	Pittsburgh Sleep Quality Index	✓			✓	✓	✓	
**Cognitive/psychological**	Spielberger Trait Anxiety Inventory	✓		✓	✓	✓	✓	
	Perceived Stress Scale	✓		✓	✓	✓	✓	
	Quality of Life SF-12 with RAND score	✓		✓	✓	✓	✓	
	Happiness Scale	✓		✓	✓	✓	✓	
	Edinburgh Postnatal Depression Scale	✓		✓	✓	✓	✓	
	Pregnancy & Weight Gain Attitude Scale (PWGAS), modified	✓						
**Socio-cultural**	Weight gain advice and purposeful weight control attempts survey	✓			✓	✓	✓	
	Social Support	✓		✓	✓	✓	✓	
	Life Events	✓		✓	✓	✓	✓	
**Biologic pathways (specimens & imaging)** ^**‡**^	Maternal Blood	✓	✓	✓	✓	✓	✓	
	Umbilical Cord Blood		✓					
	Placenta		✓					
	Umbilical Cord		✓					
	Adipose Tissue^†^ (Subcutaneous & Omental)		✓					
	Magnetic Resonance Imaging^†^			✓				
	Mouthwash Buccal Epithelial Cells for DNA	✓					✓	
**Weight outcomes**	Maternal Weight	✓	✓	✓	✓	✓	✓	
	Neonatal Weight & Length		✓	✓	✓	✓	✓	✓

*Baseline visit occurring in pregnancy at approximately 28–34 weeks gestation.

^†^Subset of subjects only.

^‡^Participation in biologic specimen component of study was optional.

#### Demographic

Baseline demographic data are collected on the day of enrollment by study personnel through personal interview. Demographic factors of interest include age, race/ethnicity, educational attainment, marital status, occupation, work status, insurance type, household size and income and other housing-related factors. Demographics with potential for change over time are queried at each postpartum visit.

#### Biological (Including Medical History, Clinical Assessments, Anthropometric Data and Genetics)

Baseline medical history is collected on the day of enrollment by study personnel through personal and medical record review. Characteristics surveyed include age at menarche, parity, interval from last pregnancy and obstetric history including prior complications like gestational diabetes, hypertensive disorders of pregnancy, prolonged bedrest and others. Medical chart review is performed to confirm or clarify aforementioned information and to retrieve information on medication use, pregnancy dating, and weight-related services such as nutrition consultations. *Estimated delivery date* and thus gestational age is determined by chart review, based on 1st trimester ultrasound or clinical dating that agrees with 2nd trimester ultrasound [49].

*Blood pressure* is measured three times in the right arm after 15 mins of sitting [50] and with a 15 minute lapse between each measurement, using an automated Dinamap XL blood pressure monitor [51]. *Urine* is evaluated for the presence of *protein or glucose* using Siemens multistix 10SG reagent strips for urinalysis.

*Height* is measured using a 1 Seca 213 Portable Stadiometer. *Prepregnancy weight* is self-reported at first prenatal visit and abstracted from prenatal record. *Pre-pregnancy body mass index (BMI)* calculated as prepregnancy weight (kg)/height^2^ (meters^2^). BMI considered continuously and categorically as per World Health Organization criteria: underweight (<18.5 kg/m^2^), normal (18.5-24.9 kg/m^2^), overweight (25.0-29.9 kg/m^2^) and obese (≥30 kg/m^2^) [3]. In accordance with prenatal care standard, weight and gestational age at each visit is recorded from digital clinical scales and was abstracted from records.

*Skinfold thickness (SFT)* measurements are performed at seven body sites (biceps, triceps, subscapular, iliac, costal, mid thigh, lower thigh), on the subjects’ right side with a calibrated Harpenden skinfold caliper (British Indicators, Sussex, UK), by methods and placements as described by Huston-Presley et al. [52], to quantify tissue distribution. All skinfolds are assessed three times at each study time point; a mean value of the three is computed. In cases where two measurements differ by more than 1.0 mm, the skin fold is measured a fourth time and the mean value of the four values is averaged.

*Body circumferences* (upper arm, waist and hip) are obtained recognizing that waist measurements are limited in pregnancy and the immediate postpartum period. Two waist circumferences are measured: (1) midway between the lowest lateral border of the ribs and the top of the iliac crest as per WHO recommendations and (2) at the top of the iliac crest as per NIH standards, in relaxed subjects during expiration [53]. Hip circumference is measured at the maximum circumference overlying the buttocks. Arm circumference measured midway between the axilla and the elbow. All circumferences measured 3 times and an average calculated.

*Biologic specimens* are collected over the course of the study with the goal of gaining insight into biologic pathways and epigenetic signatures affected by or contributing to weight outcomes. As the main aims of this study did not focus specifically on biomarkers and biosignature evaluation, but rather the acceptability of serial biospecimens collection, subjects could opt-out of contribution in part or completely.

*Maternal venipuncture* for blood collection is performed at each study visit for consenting subjects. Additionally, at the time of delivery, *umbilical cord blood* is collected after delivery of the baby but prior to delivery of the placenta. All plasma samples are aliquoted into 1 ml cryovials and stored at −80°C until assayed with freeze/thaw cycles limited to a maximum of two. All samples are analyzed in duplicate according to manufacturer instructions by a single investigator (NNZ) on the same day to minimize day-to-day variation. Samples are measured using a commercially available MagPix Milliplex® kit (EMD Millipore) with a minimum of 100 positive beads for each assay acquired with Luminex Magpix laser-based fluorescent analytic test instrumentation (Luminex Corporation, Austin, TX). Manufacture supplied controls are used to monitor coefficients of variation (<15% and 20% for intra-assay and inter-assay, respectively). Samples are diluted as per manufacturer instructions. Serum evaluations include adiponectin, c-reactive protein (CRP), interleukin-1b, interleukin-6, interleukin-8, insulin, leptin, PAI-1 total, and TNFα.

After delivery of the placenta, a 1x1cm segment of *placenta* and a 1 cm length of *umbilical cord* are placed in 4% para-formaldehyde and a second similar sized specimen of each are placed in normal saline. Prior to study completion, a single mouthwash sample of *buccal epithelial cells* is collected with Scope brand mouthwash [54].

For the subset of subjects who have Cesarean delivery for obstetric indications, and who consent, two 1x1 segments of *omental (visceral adipose) and subcutaneous adipose tissue* are sampled after the uterus is returned to the abdomen, and after the rectus fascia is re-approximated but prior to skin closure, respectively. From each site, one sample is placed in 4% para-formaldehyde and one in normal saline. The size and number of adiopocytes within the samples is determined by light microscopy. Capillary density is measured by counting number of cell profiles stained with endothelial specific markers (e.g. vWF) in 10–20 fields per biopsy specimen.

To quantify adipose deposition (visceral vs. subcutaneous) following maximal GWG [55], a subset of eligible participants (whom either did not have a Cesarean delivery or whom had subcuticular stitches for skin closure at the time of Cesarean, and whom have no metal foreign bodies), are asked to undergo optional *magnetic resonance imaging (MRI)* during the 2–4 day postpartum inpatient stay. Images are captured centrally which decreases effect of postpartum fluid shifts that most often settle peripherally in dependent regions.

#### Behavioral

At each of two study time points, *dietary intake* is assessed using three telephone-administered 24-hour (24HR) dietary recalls (two weekdays and one weekend over a three-week window), with each call duration lasting 15–30 minutes. The first dietary assessment is conducted in pregnancy at baseline, and then again at the three-month postpartum assessment. 24HR dietary recalls are considered the gold standard dietary assessment method for population-based research, with three recalls per time point being appropriate to examine inter- and intra- dietary variation patterns [56]. These assessments are administered by trained dieticians who elicit information on dietary intake utilizing the multiple-pass technique, allowing for several distinct opportunities to obtain information about participants’ food intake during the previous 24 hours. This approach, and associated software, allow for range and logic error checking, information prompting and corrections. 24HR recalls are analyzed using the University of Minnesota Nutrition Coordinating Center's (NCC) Nutrition Data System for Research (NDS-R-2010) software (updated annually) [57]. The system consists of data entry and analysis software and comprehensive food nutrient databases. The database contains over 18,000 foods (including culturally unique foods) and 8,000 brand name products along with values for 155 nutrients, nutrient ratios and other food components, allowing for analysis of individual dietary variations including portion size, preparation methods, ingredients, assessments and timing of eating. A Dietary Supplement Module captures supplements used by participants.

*Physical activity* is assessed on the same phone call via a validated 24HR physical activity recall [58,59]. Similar to the dietary recalls, three physical activity recalls are administered at baseline (in pregnancy) and then at three months post-partum, each lasting 5–10 minutes. Detailed information about specific types and intensities of activities are summarized and metabolic equivalents (METS) are calculated using Ainsworth and colleagues compendium of physical activities [60].

Additionally, the Pregnancy and Physical Activity Questionnaire (PPAQ) [61], modified for administration in the post-partum period, is administered by research personnel at all assessment time points. The PPAQ is a self-administered measure of physical activity that has been validated using ActiGraph accelerometers. This measure provides physical activity data in various domains relevant to pregnant and postpartum women, including occupational activity, household/care-giving activities, and sports/exercise [61].

Extent of *breastfeeding, breast pumping, formula and other infant feeding practices* are assessed with select questions from the Center for Disease Control’s (CDC) maternal-infant feeding study [62] and a modified version of the United Kingdom’s Medical Research Council Epidemiology Unit’s feeding and growth questionnaire [63]. Questions probe for exclusivity of breastfeeding versus extent to which other liquid and food products are offered to infants so as to quantify maternal energy expenditure with regards to lactation efforts.

*Sleep quantity and quality* are assessed with the 19 item Pittsburgh Sleep Quality Index [64], which has been used to assess sleep disturbance in pregnant [65] and post-partum [66] women.

*Tobacco and alcohol use* are queried at each study visit and recorded. Selected items from the Behavioral Risk Factor Surveillance System questionnaire [67] are utilized to assess tobacco.

#### Cognitive/Psychological

Self-administered survey tools are used to assess psychological and cognitive/attitudinal variables at all study time points. Areas assessed include anxiety evaluated by the 20-item Spielberger State Anxiety Inventory(SSAI)) [68], depression as measured by the the 10-item Edinburgh Postnatal Depression Scale (EPDS) [69,70], stress as assessed by the 10-item Perceived Stress Scale [71], a measure of life events [72], and quality of life assessed via the SF-12 [73]. We also assessed positive affect using the Happiness Scale modified (4 questions) [74]. Attitude towards pregnancy and weight gain were assessed using the modified Pregnancy and Weight Gain Attitude Scale (18 questions) [75] and questions on pregnancy intendedness. Selected eating behaviors namely emotional eating, uncontrolled eating and cognitive restraint are evaluated via the 18-item Three-Factor Eating Questionnaire [76], and a 28-item inventory assesses food cravings [77].

#### Sociocultural

Sociocultural survey assessments are completed at each study time point and predominantly evaluate overall social support using the 20-item Medical Outcomes Study (MOS) social support survey [78] and pregnancy-specific social supports [79-81] (Lobel: The prenatal social support instrument (PSSI), unpublished).

### Weight outcomes

*Total gestational weight gain* (GWG) is calculated by subtracting self-reported pre-pregnancy weight from weight at time of delivery admission or documented weight at last prenatal visit. GWG is considered continuously and categorically (inadequate, appropriate or excessive, as per IOM-recommended ranges) over the course of gestation and at delivery. Categorical GWG assessment is BMI-specific and accounts for gestational age at measurement as per IOM recommendations [82]. Minimum and maximum gain at each gestational age week is calculated based on recommended velocities of gain in first trimester (ending week 13) and throughout second and third trimester assuming the following gain ranges achieved by 40th week: 28–40 lbs for women of underweight prepregnancy BMI, 25–35 lbs for normal, 15–25 lbs for overweight, and 11–20 lbs for obese gravidas [3].

*Postpartum weight loss or retention* is calculated at 6 weeks, 3 months and 6 months postpartum by subtracting measured weights at these study visits from measured weight at last prenatal care visit. All study specific weights are measured three times on a digital scale (TANITA BWB-800AS) and averaged.

Infant weights are measured in order to assess associations between maternal variables and infant weight. Infant weights are measured at all postpartum assessment time points when the infant is in attendance with maternal subjects, utilizing a Tanita BD-590 pediatric scale. Additionally, pediatrician records are obtained once each child reaches a year of life to capture length, weight and head circumference measurements for the offspring of each maternal participant from birth through one year.

#### Participant safety

To ensure the safety of participants who might be experiencing mood disorders, the EPDS is scored immediately after completion and before the participant leaves the study office. Participants scoring 12 or greater (suggestive of possible clinical depression) who score negative on the suicide question are encouraged to contact their provider to discuss symptoms. Participants scoring 12 or greater and with positive responses to the suicide question are considered at acute risk of injury or harm; an appropriately trained person performs a safety assessment and refers for immediate psychiatric evaluation if warranted.

### Statistical analysis, power and sample size

The primary outcomes for this study are change in weight from pre-pregnancy to last measured prenatal weight prior to delivery (i.e. gestational weight gain or change) and change in weight from last measured prenatal weight to weight at 6 months post-partum (i.e. postpartum weight change). Additional weight measures are taken at each prenatal visit and at 6 weeks and 3 months postpartum. One of the main purposes of this study is to collect data related to these outcome as there is limited data in the literature thus we used standard deviation units as a way to determine a reasonable sample size. Assuming the definition of the primary outcomes from above and using a paired t-test for the main analysis with a two-tailed alpha = 0.05 and power = 0.80, we are able to detect a difference of as little as 0.30 standard deviation units with 100 patients. Adjusting for potential drop-out or missed data of 10%, we planned recruitment of 110 patients for either change in weight outcomes. To estimate detectable associations between predictors and either change in weight outcomes, we will use correlations since regression coefficients are essentially standardized correlations. So, for correlations between predictors and outcomes, we will have power = 0.80 to detect correlations of 0.28 with 100 patients in a regression model (as described below). In fact, because we will use multivariate regression models to partition the overall variance, we expect to have more power than estimated.

Descriptive statistics will be calculated in the usual way. To estimate the gestational weight change and the post-partum weight change, we will first calculate the change in weight as indicated above and use a standard paired t-test (if the weight change is normally distributed) or a Wilcoxon non-parametric test (if weight change is not normally distributed) to determine if there is a significant change in weight from pre-pregnancy to last prenatal visit preceding delivery weight and from this to six months postpartum. To determine the effect of the various predictors on these outcomes, we will use two approaches to model construction. First, we will use general linear models to model change in weight during gestation or change in weight post-partum, using the definitions for these outcomes from above. Second, we will use a mixed effects model to examine the individual trajectories of change over time, using all of the measures of weight throughout the gestation and post-partum periods. The predictors will be similar for the two analytic approaches, based on the conceptual model in Figure 1. In general we will first investigate the effect of related groups of predictors (such as the behavioral predictors from Table 1) and then combine the resulting significant factors into the final model. With a relatively limited sample size, this will be a feasible approach – and potentially more revealing – than a more omnibus variable selection process.

## Results

Demographic characteristics of the study sample (n = 110) are presented in Table 2. As anticipated, the study population is relatively healthy and consistent with the general population seeking prenatal care in the faculty and resident ambulatory obstetric clinics that constitute the study sites. The mean age is 28.3 years, with a mean pre-pregnancy BMI of 25.7 kg/m indicating that a majority of the women were overweight or obese prior to becoming pregnant. The mean gestational age at recruitment was 32.5 weeks. One-third of the cohort is non-white, over a quarter are Latina/Hispanic, 15.5% report a primary language at home other than English, and almost a quarter are foreign born. The majority of participants are multigravida, are high school graduates or have higher levels of education, and work outside of the home. Almost half of the sample reported household financial challenges.

**Table 2:** **Cohort demographic characteristics**

Demographic characteristics	Mean	SD	Median	Interquartile range
**Age (years)**	28.3	5.1	28	24-32
**Gestational age @baseline study visit (weeks)**	32.5	1.8	32.6	31.4-33.7
	**N**		%*	
**Prepregnancy BMI (kg/m** ^**2**^ **) (n = 110)**				
Underweight	1		0.9	
Normal	52		47.3	
Overweight	32		29.1	
Obese	25		22.7	
**Race (n = 93)**				
White	63		67.7	
Black/African American	7		7.5	
Asian	5		5.4	
Hawaiian/Pacific Islander	1		1.1	
Other	11		11.8	
Multiracial	6		6.5	
**Ethnicity (n = 99)**				
Not Hispanic, Latino or Spanish	71		71.7	
Hispanic, Latino or Spanish origin	28		28.3	
**Non-U.S. born (n = 99)**	24		24.2	
**Non-English primary home language (n = 98)**	16		15.5	
**Multigravida (n = 108)**	75		69.4	
**Relationship**				
In relationship with father of baby (n = 98)	92		93.9	
Married (n = 99)	60		60.6	
**Education (n = 99)**				
≤8th grade	2		2.0	
>8th grade, < high school	3		3.0	
High school graduate or GED	20		20.2	
Trade or technical school after HS	5		5.1	
Some college	25		25.3	
4 year college degree	23		23.2	
Other	21		21.2	
**Employment (n = 100)**				
Working full or part-time outside home	65		65.5	
Homemaker	15		15.0	
**Household ability to make ends meet (n = 100)**				
Great difficulty	12		12.0	
Some difficulty	36		36.0	
No difficulty	52		52.0	
**Location of care (n = 109)**				
Resident practice	34		31.2	
Faculty practice	75		68.9	

*Percentiles based on total number for whom data available and does not account for missingness.

## Discussion

Maternal and childhood obesity are significant public health issues that affect general immediate and long term health of women and children. Women receive more medical attention in pregnancy and the postpartum period than at any other healthful time in their lives; this time is ripe for intervention, especially as women are generally motivated to improve their health for benefit of their offspring. However, this is also a time of significant adaptations as it relates to physical, physiological, hormonal and behavioral changes (including sleep, diet, physical activity, smoking habits), new and different stressors, mood fluctuations, and changes in sociocultural identity and responses from the social environment, along with numerous other weight relevant factors. Excessive pregnancy weight gain and lack of postpartum weight loss [9,83] are associated with maternal obesity which increases the risk of diabetes, cardiac disease [83], some cancers [16], and other health consequences. Maternal obesity and excessive pregnancy weight gain are associated with childhood obesity [3] and its downstream effects which are varied and include metabolic dysfunction, asthma, and behavioral disorders [84]. The critical interplay of socioeconomic issues and weight-related behavioral and other patterns [84] are infrequently or inadequately addressed in weight interventions to improve maternal and offspring health. Comprehensive examination and understanding of behavioral, social, cultural and environmental contexts through a lifespan biopsychosocial model is critical to the design of future interventions with the potential to substantially and effectively improve maternal and child health.

This study is grounded in theory as conceptualized by women’s weight trajectories during both pregnancy and the post-partum period, and long-term maternal and child health and well-being, within a lifespan biopsychosocial framework. This study was designed to determine the feasibility of understanding multifactorial influences on maternal pregnancy and postpartum weight trajectories with the goal of gaining critical foundations of knowledge, experience and infrastructure on which to base future intervention research. Large trials recruiting socioeconomically and racially/ethnically diverse women are necessary to identify and analyze moderators and mediators of weight gain and loss as they relate to pregnancy and the postpartum period, and elucidate their effects on maternal and offspring health. We piloted critical variables that are relevant to future large scale studies addressing these issues.

The strengths of this study protocol are several. This is a longitudinal study design that includes a large number of variables and comprehensive assessments. Each of these critical factors individually and/or in aggregate examines different contexts of the biopsychosocial model. We were able to recruit a diverse population of women with 28% of our subjects being Latina, almost a quarter being non-US born and with approximately 15% from households speaking primary languages other than English. The study was able to recruit a socioeconomically diverse sample, as evidenced by their range of abilities to meet their household needs. This is critical for analysis of interactions amongst the biologic, psychologic, and social variables. The subject population represents women of childbearing age with more than half being overweight or obese in pregnancy [85], including one out of 5 being obese [86].

A significant strength is the pilot nature of this study – performing a pilot acknowledges the importance of determining the feasibility of and acceptance of the design and overall burden to targeted participants. This is critical before embarking on a larger scale endeavor. However, our relatively modest sample size is a limitation. Adequately powering an intervention study with consideration of mediators and modifiers will require a larger sample size and is beyond the scope of this project. Generalizability of study findings may be limited by exclusion of non-English speakers, women with pre-existing physical and mental health conditions, adolescents, and women who did not seek care in early pregnancy.

Evidence of feasibility, acceptability, and consideration of weight influencing factors and behaviors other than diet and physical activity are essential to future epidemiological and intervention studies, including the design of new interventions that optimize appropriate maternal gestational weight gain and subsequent postpartum weight loss. We anticipate that the current study will produce valuable data and insights to help guide application of these findings to interventions targeting maternal health for maternal benefit but also for offspring benefit, thus promoting intergenerational health.

## Abbreviations

BMI: Body mass index
DOHad: Developmental origins of health and disease
EPDS: Edinburgh postnatal depression scale
GWG: Gestational weight gain
HIPAA: Health Insurance Portability and Accountability Act
IOM: Institute of Medicine
NIH: National Institute of Health
PPODS: Pregnancy and postpartum observational determinants study
vWF: von Willebrand’s Factor
WHO: World Health Organization
